# Affordances of musical instruments: Conceptual consideration

**DOI:** 10.3389/fpsyg.2022.974820

**Published:** 2022-11-16

**Authors:** Markus Tullberg

**Affiliations:** Music Academy of Malmö, Lund University, Lund, Sweden

**Keywords:** affordances, musical instrument, music education, enactivism, ecological psychology, Material Engagement Theory, Skilled Intentionality Framework

## Abstract

While the concept of affordances has been applied in music research, it has not been satisfyingly developed regarding musical instruments. The resulting vagueness restricts the potential of the concept to guide exploration, discussion, and development of new approaches towards musical learning. Also, the concept of affordances comes with strong ontological claims and thus prompts the researcher to be careful when merging it with other theoretical domains or applying it in empirical studies. Consequently, the present article aims at contributing to a conceptualization of affordances of musical instruments by highlighting and discussing components that are necessary to consider in such a project. The first part consists of an overview of key elements of ecological psychology and more recent theoretical contributions, which are of relevance to the aim of the article: Material Engagement Theory, Skilled Intentionality Framework, and Sensorimotor Contingency Theory. A brief review of examples on how the concept of affordances has been applied in music research is presented. The main section of the article discusses four components, vital to further theoretical developments on affordances of musical instruments: the musical niche, spatial networks, sensorimotor relationship, and the amodal nature of affordances. Central to the argument is an understanding of affordances as relational, limited in scope and bound up with the physical interaction between musician and instrument. Accordingly, it is proposed that analytical focus in studies of musical instruments should be the sensorimotor relationship, spatiotemporally unfolding through a musical event. The article is concluded with comments upon educational implications of the presented perspective and suggestions on further research on the topic.

## Introduction

Ecological psychology and its key concept of affordances has been applied to music research (examples include Folkestad, 1996; DeNora, 2000; Clarke, 2005; Godøy, 2010; Nilsson, 2011; Menin and Schiavio, 2012; Windsor and de Bézenac, 2012; Akoumianakis, 2013; Coessens and Östersjö, 2014; Krueger, 2014; Schiavio, 2014; Duby, 2019; Koszolko, 2019; Clarke, 2020; Duinker, 2021; Tullberg, 2021; Cross, 2022). However, while literature within the field shows a variety of understandings, interpretations and applications, the concept has not been satisfyingly defined with regards to musical instruments. Confusion and vagueness surrounding the concept restraints its analytical potential to explore, describe and explain the complex ways that musicians interact with their instrument. A central issue seems to stem from the differences regarding analytical focus and scope following from the various available definitions.

The aim of this article is to contribute to a conceptualisation of affordances (Gibson, 1979/2015) of musical instruments. I do this by bringing forth and discussing components necessary to consider when applying the concept in studies on musician-musical instrument interaction. I refer to theories that support and refine Gibson’s ideas in relation to the topic, and which seems helpful in future work along these lines.

The first section recapitulates key elements of Gibson’s work, which are central to the present article, as well as relevant theoretical contributions by other scholars, sharing the same concerns as Gibson. The second section consists of a brief review of how the concept of affordances has been applied in music research. The third section discusses four conceptual components; the musical niche, spatial networks, sensorimotor relationship, and the amodal nature of affordances. I close the article by commenting upon possible educational implications.

## Affordances and socio-materiality

Gibson’s ecological psychology is best understood as a reaction towards cognitivist views. As such, it is the first fully-fledged situated theory of perception and cognition (Heras-Escribano, 2019). The concept of affordances, being a central piece of Gibson’s larger theoretical framework, cannot be abstracted from the foundations of this radical position without losing its potential to spur rethinking of perception, action, and cognition.

Among these foundational claims is the idea that perception does not rely on mental representations, memories, or internal maps (Heft, 2020). On the contrary, necessary information for the agent’s actions is available directly, without any inferences. This information includes *invariants* – consistent patterns of information that, through perceptual action, reveal the structure of the surrounding environment or the object. This interwoven relationship between action and perception includes overt movements conducted in order to manipulate objects, as well as how things are brought into focus by means of attention.^1^ The concept of affordances refers to the opportunities for action that emerge in and though these perceptual actions. Rooted in an embodied and situated perspective, affordances are related to the agent’s capabilities, needs and interest. As described by Michaels and Carello (1981): “humans do not perceive chairs, pencils, and doughnuts, they perceive places to sit, object with which to write, and things to eat” (p. 42). Affordances are thus inherently meaningful. Moreover, affordances cannot be studied or discussed without addressing the context in which an agent exist, what Gibson (1979/2015) refers to as an *ecological niche*. A niche includes the surrounding space, seen through the behaviour of its inhabitant. Thus, several niches may overlap in terms of physical space but since a niche exists in tandem with the agent, they may still be separate in terms of affordances.

However, ground breaking these ideas were, Gibson’s writings have been noted to be confusing and contradictory (Chemero, 2003; Käufer and Chemero, 2015). Hence, there are many different interpretations and applications of his concepts. Specifically, I would like to clarify my position regarding two issues.

The first point is the question whether affordances are to be understood as (i) *dispositional* or (ii) *relational* (Heft, 1989; Chemero, 2003; Stoffregen, 2003; Heras-Escribano, 2019; Magri, 2019). This question has consequences when it comes to the unit of analysis. A dispositional view leads towards studies on properties and dispositions.^2^ Instead, the relational interpretation – the position I support in this paper – implies that the continuous agent-environment system will be the locus point of analysis.

The second point concerns sense modalities. Beyond his work on visual perception (Gibson, 1979/2015), Gibson also discussed other sense modalities in terms of perceptual systems (Gibson, 1966). However, as noted by Stoffregen et al. (2017), Gibson’s argument can be understood as one irreducible perceptual system, through which the senses operate. Affordances are thus perceptual content of the *global array,* since “behavior always requires control relative to referents that cannot be specified in patterns that exist within any single type of ambient energy” (Stoffregen et al., 2017, p. 166). Accordingly, affordances are to be considered as amodal in nature.

Gibson’s work is part of, and has contributed to, a broad movement that views cognition as non-representational, situated, and embodied.^3^ Among the many noteworthy later contributions are Malafouris’ (2013) *Material Engagement Theory* (MET). Being a cognitive archaeologist, Malafouris draws inspiration from extended and embodied perspectives on cognition. Based on this, Malafouris (2013) proposes a hypothesis of the constitutive intertwining of cognition and material culture. Throughout the book, Malafouris presents evidence of the historical entwinement between material culture and human thinking and shows how the human mind has been shaped – and continues to be shaped – by objects and tools. MET holds that cognition emerges in the transactional space between actor and object, and that symmetrical relationship cannot be reduced to isolated components (Alessandroni and Malafouris, 2022).

Another important contribution is the *Skilled Intentionality Framework* (SIF), developed by Rietveld and colleagues through a series of publications (see for example Rietveld et al., 2018; Yakhlef and Rietveld, 2020; Bruineberg et al., 2021). Through Wittgenstein’s notion of *form of life,* SIF takes affordances as communal and relevant to either a species or a particular socio-cultural practice (Van Dijk and Rietveld, 2017). As such, affordances are normative. This, so called *zoomed-out perspective* deals primarily with regular and stable patterns of standing practices. The *zoomed-in* perspective on the other hand focuses on the lived experience of a local agent, negotiating a landscape of affordances in flux (Van Dijk and Rietveld, 2017). Affordances can thus be both small-scale (inviting a confined action with immediate feedback) and large-scale (unfolding in a wider time span and part of a complex project; Van Dijk and Rietveld, 2020). This double perspective is a result of a strive to bridge a gap between two sides of ecological psychology: one dealing with affordances emerging in material engagement, the other exploring social coordination (Van Dijk and Rietveld, 2017). I believe studies of affordances of musical instruments are rooted in a similar position since creative processes are as much bound up with material affordances as of cultural (Clarke, 2020).

Beyond MET and SIF, I utilize ideas from enactive researchers.^4^ O’Reagan and Noë (2001) take their point of departure in the perception-action cycle, foundational for ecological psychology. Their concept *sensorimotor contingencies* (SMC) refers to the rules of structural correspondence between motor actions and sensory changes (O’Reagan and Noë, 2001). Patterns of sensorimotor contingencies become familiar to the agent through a history of interaction with the environment, giving rise to *sensorimotor skills*. Such skills are used both in trivial tasks (e.g., opening a door handle) and in fields of expertise (e.g., playing a violin). To some degree, sensorimotor tasks are conducted subconsciously. However, we may become aware if our attention is captured or if we chose to attend to our actions, for example where we put our feet to avoid a puddle on a rainy day. Being familiar with an object, it is possible to be visually aware of things not present at the moment (Noë, 2004). I cannot see the back of the computer screen on my laptop. Yet, I know what it looks like and I have access to it if I so desire. Similarly, I do not have the flute beside me on my desk, but still I have a visual awareness of it if I chose to attend to it. In a later publication, Noë (2012) refers to this phenomenon as *presence-in-absence*.

Beyond visual perception, the theory of SMC has been applied on aural perception (Froese and González-Grandón, 2020) and tactility (Di Paolo et al., 2017). The latter group of researchers also proposes the concept of *sensorimotor schemes*. These are patterns of coordination we act through when we confront a task. Sensorimotor schemes are both a matter of perception and action. Or rather, one presupposes the other. The concept of sensorimotor schemes also includes a dimension of normativity, since motor actions generally are more or less successful, according to intentions.

## Affordances and music research

Among the disciplines of music research, different definitions of affordances are offered. One of the points on which interpretations are diverse, is the scope of the concept. Can affordances be applied to possible actions beyond the immediate situation, or should the concept be reserved for sensorimotor interaction in an unfolding event? The lack of consensus regarding implicit and explicit answers to this question has led to affordances being characterized by an “*epistemological vagueness*” (Schiavio, 2014, p. 85). While not offering a complete review of the use of the concept of affordances in music research, some examples are needed in order to position the present paper in the research landscape.

The divide between two sides of ecological psychology highlighted by Van Dijk and Rietveld (2017) can be used to discern different approaches to the concept of affordances in music research. One example of the focus on material engagement is Folkestad’s (1996) study of children composing music on computers. In this study (to my knowledge the first to apply the concept of affordances in music), affordances provided a way to discuss the possibilities of computer software as these were used by the children. Interestingly, children without prior experience of playing instruments showed a more exploratory approach towards the software than those with musical background, whos’ perspective and expectations on the computer were more in line with those on traditional acoustical instruments. As Folkestad (1996) notes, the perspective of affordances implies a definition of creative action as being an ability to perceive and utilize new affordances.

The focus on a social dimension can be exemplified with DeNora’s (2000) study on the role of music in people’s everyday life. In her words, “music’s affordances – moods, messages, energy levels, situations – are constituted from within the circumstances of use” (DeNora, 2000, pp. 43–44). Examples following this interpretation of affordances include a study on a music education outreach project in Australia (English et al., 2021).

Exploring ecological psychology and musical listening, Clarke (2005) proposes a “reverse” understanding of perception of musical meaning. While traditional, cognitivist perspective on auditory perception builds on a bottom-up approach (parameters such as rhythm, pitch, and intervals are perceived and the musical meaning is constructed in the brain of the listener), the ecological approach according to Clarke (2005) claims that musical meaning is perceived directly. As Clarke shows, this meaning can be discussed in terms of affordances. In a similar vein Krueger (2014) adapt the concept of affordances in his exploration of musical listening and emotional regulation. Krueger combines the extended mind hypothesis (Clark and Chalmers, 1998) with music’s ability to scaffold affective states. Within this framework, the concept of affordances is used to highlight the relational character between the agent and the surrounding environment.

The concept of affordances is also taken as analytical focus in studies on certain aspects of musical practice, such as gestures (Godøy, 2010; Duby, 2019) and musical structures (Duinker, 2021). Norman (1998) transformed the concept in his work of design theory. Through this work, affordances found its way into digital design. Studies within these fields are commonly using an interpretation of the concept that relies more on conventions of communication than on ecological psychology (Heras-Escribano, 2019). As such, the concept of affordances can be found in music research concerning digital technology. Examples include Koszolko’s (2019) study in digital tools in music production and Akoumianakis (2013) study on music lessons depending on computer software. In a recent paper, Cross (2022) explores interactive affordances as properties of rhythm and pitch in music and speech, emerging in the interaction between humans.

While the concept of affordances has been applied in music research to some extent, examples of research with an explicit focus on musical instruments are more scarce. A dispositional understanding of affordances in this context is close to the notion of *idiomaticity*: “Whether one has the adequate effectivities or chooses to attend to them or not, the instrument does come with a set of carefully designed affordances which guide exploration and constrain action” (Windsor and de Bézenac, 2012, p. 8). A contrasting view can be discerned in the writings of Coessens and Östersjö (2014): “an instrument affords different musical possibilities to different performers; hence, the affordances of an instrument are as dependent on the individual performer as on the acoustic properties of the instrument” (p. 337). Along the same lines, Clarke (2020) states that “the creative process emerges from what a physical instrument affords to the specific body of a particular player” (p. 14). Menin and Schiavio (2012) articulates a similar view:

A skilled guitarist might be unable to say where to put her/his finger to perform a solo, but s/he can use the motor knowledge of the fingers to reconstruct the actual set of notes played, by just putting the hand on the strings. We believe that this sensory-motor process not only represents the basis of musical understanding, but it can also shed light on the notion of musical affordance, relying on a sub-cognitive, pre-linguistic, intrinsically motor form of intentionality. (p. 210)

The above quote is a good point of departure for the next section, which discusses components that I find essential to consider in further conceptual work on affordances of musical instruments. I focus on a zoomed-in perspective which takes the sensorimotor relationship between musician and instrument, situated within a musical niche, as a spatiotemporal transactional space and analytical focus point.

## Conceptual considerations

### The instrument and the musical niche

The concept of affordances is part of a situated understanding of cognition, and even with a definition of affordances limited in scope, environmental conditions must be taken into consideration (Clarke, 2020). These conditions are constituted by genre-specific elements, such as aesthetic value systems, institutional framings, historical background, function of the music at hand, its role in society, and its acoustic dimension. Taken together, such conditions form, what can be thought of as a *musical niche*, in which the musician-instrument relationship is situated.^5^ One of these conditions – the acoustic dimension – is omnipresent and constitutive of any musical event. Sound projects from the instrument and returns to the musician. Thus, the surrounding space is embedded in the perception-action cycle. Accordingly, the acoustic dimension needs to be considered as a component of any given musical niche.

Starting with a zoomed-out perspective, it is necessary to take into account the historical axis of the acoustic properties of a musical niche.^6^ Particular instruments require certain acoustic conditions, and vice versa: changes of performance contexts are driving developments of musical instruments (Townsend, 2020). Emerging music ideals put pressure on instrument developments. For example, new harmonic structures brought with it a demand to be able to play in different keys within the same piece. New ensemble settings invited players, and thus instrument makers, to strive for a larger sound (Powell, 2002). In short, the current standing practices have in part emerged through a co-evolution of instruments, aesthetics, and acoustics.^7^

Zooming in, acoustic properties of a certain space are not always noticed or brought to a musician’s attention. Extreme situations can however highlight the effect of acoustic feedback. Performing or recording music in an anechoic chamber not only impacts the reflection of sound, but also the experience of performing and thereby the performance itself (Freiheit, 2010; Autio et al., 2021). My own experience of playing flute inside an anechoic chamber was a physically (and musically) very unpleasant and “unreal” experience. Although nothing happened with the instrument as a physical object, the instrument felt numb. Also recording studios with dry acoustics may influence how an instrument is played. An example of the opposite provided by Östersjö (2020) in his description of performing a guitar piece in a room with a reverberation time of thirty seconds. Although both cases are on the extreme ends of a continuum, musicians continuously adjust their playing to meet the acoustic properties of the performance space (Meyer and Hansen, 2009; Kalkandjiev and Weinzierl, 2015; Steensgaard Gade, 2015; Tullberg, 2021). Furthermore, Steensgaard Gade (2015) states that if sound in a concert hall improves over times, it is probably because musicians learn to adjust their playing to the space. Beyond their playing technique, musicians today negotiate the acoustic properties of their musical niche through amplification, instrument manipulation, or by controlling the environment (Ryan and Gallagher, 2020; Segundo-Ortin and Heras-Escribano, 2021). In Nilsson’s (2011) words: “the system self-adjusts in order to optimize its resonance with the environment” (p. 123). In short, musicians’ musical lives are embedded in different acoustical realities depending on their musical niche. Whether it is a jam session, recording studio, a festival stage, an outdoor dance evening, or a concert hall, the setting influences how a desirable sound is defined and achieved. Accordingly, core elements of aesthetics are linked to such acoustic conditions – both historical and current – of a particular genre. In this way, the surrounding space can be understood as an extension of the instrument and constitutive of the affordances thereof.^8^

Understanding the instrument as inhabiting a musical niche, more that its acoustic properties are interesting. A line of research within ethnomusicology explores musical instruments as bound up with its musical, cultural and social context (see for example Ronström, 1989; Qureshi, 1997; Dawe, 2001; Bates, 2012; Eriksson, 2017). From this perspective, musical instruments can be seen as “one specific acoustical aesthetic complex” (Racy, 1994, p. 51). As Kvifte (2008a) points out, the identity of an instrument is not static. A fiddle and a violin share the same physical appearance, but the two names bear different connotations. The *taragot* forms a reverse example as the name refers to two physically different instruments, sharing the same identity as Hungarian national instrument (Kvifte, 2008a). Further exemplifying with the Norwegian instrument *sjøfløyte,* Kvifte (2008a) states that “the style of music is a decisive factor to establish the identity of an instrument” (p. 47).

The perceived identity of an instrument has bearing upon how it is commonly used within a particular genre. Such normative meaning can be understood in terms of *canonical affordances* (Costall, 1997). Considering a clarinet, the most apparent canonical affordances invite individuals to hold the instrument in two hands and blow into it in order to produce a sound. However, embedded in the standing practices (Van Dijk and Rietveld, 2017) of a musical niche, is yet another normative value system in terms of its aesthetics (Alperson, 2008).

Zooming in further, it is necessary to draw a distinction between *occasion* and *event* (Qureshi, 1987). Occasion refers to a generalized performance context of a musical niche (e.g., a jazz jam session, a piano recital). As such, the notion of an occasion comprises concepts, norms, and typical acoustic conditions. An event refers to a specific performance, the unfolding of which depends on its underlying nature (the occasion), the specific space, and the actions of the present individuals.

### Instrumental space and spatial networks

The notion of space carries a multitude of meanings in relation to music. These shifting meanings can be broadly grouped into either literal or metaphorical (Di Stefano, 2022). The literal meaning refers to the sound source in physical space, while the metaphorical meaning refers to non-physical, imaginery space. Space, in both meanings is perceived, albeit in different ways. Instrumental space refers to the first (the spatial layout of the instrument), while the concept of *spatial networks* also draws upon metaphorical perceptions of space. Bielawski (1979) offers an interesting perspective on this topic, proposing a model that takes musical instruments as transformers of bodily gestures into musical gestures. By not viewing the instrument as input and output device,^9^ the analytical focus can be the interaction between musician and instrument (see for example Sudnow, 1978; Baily, 1985; Edlund, 2003; Aho, 2016).^10^

From such an interactional perspective, the musician’s hands are equally important as the spatial layout. The “sub-cognitive, pre-linguistic, intrinsically motor form of intentionality” (Menin and Schiavio, 2012, p. 210) mentioned above is not limited to musical practice, but a general characteristic of human hands. Almäng (2008) provides an example from an activity relatable to non-musicians:

I am unable to report the location of the various keys on the keyboard. If someone gave me a keyboard with the signs of the letters removed and asked me to point out where the letter ‘A’ normally is I would be unable to answer, unless I was allowed to write on the keyboard and could observe the movements of my hands. (p. 167)

Likewise, the ability to play a musical instrument relies on specific sensorimotor schemes (Di Paolo et al., 2017). In musical performance the role of the hands is not only as executors. Their movements are coupled with haptic and tactile sensations and furthermore, having coevolved with the brain they are integrated in the expressive system of human beings, in that the movements have a potential to give rise to an embodied response (Leman et al., 2017). This has implications for the experience of musical performance, since the expressive system includes a motivational, autotelic and sense-giving component dimension.

Bringing together instrumental space and the role of the hand, DeSousa (2017) devises the concept of *spatial networks*. By this he refers to perceptual content, dependent on both the spatial layout of the instrument and idiomatic conventions of the musical niche, as well as on capabilities, interests, background, and preferences of an individual musician.^11^ While the spatial layout of an instrument is generally a stable property – an invariant (Gibson, 1979/2015) – of a musical instrument, spatial networks are not. They are dependent on both the physical manifestation of the instrument and sensorimotor schemes. Furthermore, spatial networks can also be a product of theoretical concepts such as scales, chords, and recurring melodic structures. When a spatial network is integrated through sensorimotor schemes it can be said to reside in the musician’s fingers (Leman et al., 2017, p. 177). This “automaticity” allows the musician to direct attention to aspects of the performance other than the movements of the hands. However, this phenomenon necessarily obscures aspects of the playing for the musician. Ornamentation can for example be described as “a disease, spreading without control” (Tullberg, 2021, p. 171). In order to make these hidden actions available for reflection, a shift of perspective is needed. One way to accomplish this is to record oneself, since a listener perspective is distanced from the performing body (Windsor, 2016).^12^ Another path is exemplified by jazz musician Kurt Rosenwinkel’s process of retuning his guitar (DeSousa, 2017). Disrupting the spatial networks, Rosenwinkel is forced to innovation, and thus new affordances of the instrument can emerge.

Rosenwinkel plays a chord on his radically retuned guitar but claims not to know what it is. In the moment, he cannot name it or place it in a theoretical system. He cannot reduce its sensual qualities – its felt shape, its sonic color, even its specific pitches – to an internal idea. (DeSousa, 2017, p. 106)

As this quote exemplifies, spatial networks emerge through multiple perceptual processes, a cross-modal phenomenon which becomes overt when it falls apart. Given that both literal and metaphorical meaning of space (Di Stefano, 2022) are relying on perception, spatial networks can be said to blur the line between them.

### The sensorimotor relationship and the musical event

The continuous interaction with the instrument leads to development of a sensorimotor relationship with the instrument. The spatial networks are but one, although central part of the sensorimotor relationship. Playing technique, understood broadly as structural patterns of coordination, involve gestures in a wider sense (Alperson, 2008; Kim, 2020). In the case of wind instruments, the respiration system needs to adjust to embouchure and bore of the instrument in similar ways as hands adjusts to the instrumental space (Ljungar-Chapelon, 2002; Balosso-Bardin et al., 2017; Tullberg, 2021). For a church organ, the spatial networks are not limited to the hands, as also the feet are involved. In short, a musician is bound up with the instrument through the perception-action cycle and constantly reacting and adjusting to the sensorimotor contingencies according to a normative framework. A fundamental difference from a cognitivist approach is that a normative framework is not a matter of internal, mental processing, but rather a constitutive dimension of sensorimotor schemes (Di Paolo et al., 2017).

While affordances of the musical instrument are situated within this sensorimotor relationship, these emerges in an unfolding event, in Qureshi’s (1987) sense of the word. The unfolding of an event can be understood as “changes in the layout of affordances” (Chemero, 2003, p. 192). This means that the affordances of any given moment are dependent on what just has happened and on expectations on what to come next. A single note is, among other parameters, meaningful in relation to the temporal dimension. The immediate future is anticipated, but also imagined (van Dijk and Rietveld, 2020). This imagination, constrained by contextual and musical structures guides the intentional exploration of the musical event.

Taking the sensorimotor relationship as the unit of analysis also implies that musical skills are situated and fragile. Although the sensorimotor relationship is developed over time, through practice and experience, it can be subject to temporary or permanent negative change. Having a cold, for example has drastic negative impact on wind instrument playing and may also affect hearing. Injuries or tendinitis may cause long term restrains and a necessity to fundamentally adjust the fingering technique.^13^ But the sensorimotor relationship is also volatile due to changes in the other end – the instrument. Strings are bursting, keys are sticking, wood is cracking, and instruments need to be broken in. Temporary changes can also arise in the hands of the musician. Having practiced particular movements (i.e., a challenging passage) may momentarily make a musician more attuned to the spatial networks with which the musician engages (Tullberg, 2021).

In order to avoid affordances to remain “impossible, ghostly entities” (Chemero, 2003, p. 182), I present one example from my own practice (Tullberg, 2021). During an interview with Breton flute player Jean-Michel Veillon, he played a melodic phrase in which he used an alternative fingering for the tone Bb. On my request, he stopped and demonstrated the fingering, which was new to me. I reproduced the fingering and the accompanying movement (a forward nod). The pitch and timbre corresponded with what Veillon produced. He explained that he had found this fingering himself in order to enhance this particular melodic phrase. For Veillon, this alternative fingering was an affordance of the musical instrument, but for me it was not. At the moment of reproducing the fingering, it was solely an isolated piece of playing technique. For the fingering to be perceived as an affordance, something else is needed. With practice, the fingering could be integrated as a sensorimotor scheme and become part of my sensorimotor relationship with my instrument. If so, I can make use of it in a musical event.

Although this particular fingering can be described as a property of a spatial network, the dimensions of the sensorimotor relationship are interwoven. The fingering was bound up with a loosening of the embouchure and the nodding gesture, which modified the angle between the lips and embouchure hole as well as impacted the airflow. The resulting sound was a veiled tone which had the character of a wave, both in terms of dynamics and pitch. In context of the melodic phrase, the fingering certainly contributed to the expression that Veillon sought and indeed it was a distinctive example of his aesthetics.

### Perceiving affordances of a musical instrument

The sensorimotor relationship cannot fruitfully be sliced up according to traditional modalities. Such analytical approach would be a brutal abstraction of the experience of playing a musical instrument, at least from a perspective on affordances as emerging through an active exploration that involves the whole body (Noë, 2012; Stoffregen et al., 2017). One component of this active exploration is the overt movements integrated in the act of playing. Another component is attention, which can be both “caught” and intentionally directed (O’Reagan and Noë, 2001; Froese and González-Grandón, 2020). Therefore, attention is of vital importance in musical practice. An amodal understanding of affordances suggests that attention is less constrained by different sense modalities, but rather about focusing on different aspects of the unfolding event (Chemero, 2003).

An observation from a previous project (Tullberg, 2021) can serve as example. A musically rather homogenous group of flute players, including myself, met for ten two-hour sessions in a co-operative inquiry (Heron, 1996). All of us were focused on orally transmitted traditional music. Nevertheless, when we probed into the experience of flute playing, a quite diverse picture emerged when it came to how we perceived our instruments in the act of playing. Our attention was directed towards different aspects of the interaction, shifting as the musical event unfolded. There were also individual variations with regards to how the musicians in the group used their attention, both consciously and intuitively. For example, while learning a new tune, some focused on the fingering and the spatial layout of the instrument as a way to “grasp” the melody. Others were “thinking” in sound and focused on being able to repeat the melody either by singing or through *musical imagery* (Bailes, 2007; Huovinen and Tuuri, 2019). When we experimented with musical tasks such as transposition and improvisation, some of the participants were able to access spatial networks of other instruments, not present in the room. Flute players with experience from instruments such as guitar and piano could be “visually” guided by the structures of chords of these instruments. These structures could be transferred to the flute in the moment of playing. This is an example of what Noë (2012) refers to as *presence-in-absence*.^14^ Such findings imply that musicians interact with their instruments through habit as well as active exploration through attention.^15^ Since affordances emerge through active perception, these are partly defined through how a musician consciously directs his or her attention.

Furthermore, affordances are skill-relative (Noë, 2004) and emerging expertise develops perception, specific to the field of practice. This cultivated attunement to the fine-grained nuances of the interaction with the instrument has the potential to become an integral part of the body and the perceptual apparatus (Froese and González-Grandón, 2020). In terms of sensorimotor relationship, a trumpet in the hands of a beginner is a different instrument from a trumpet in the hands of a professional musician. They both may hold the exact same instrument in their hands but the perception of it will be very different. This is not due to subjectivity and a matter of perspective, but a matter of an embodied, physical reality and a process over time of adjusting to an instrument. Accordingly, the concept of sensorimotor relationship is one way to explain the collapsed dichotomy between the subjective and the objective, that one of Gibson’s (1979/2015) most often quoted passages is referencing: “an affordance is neither an objective property nor a subjective property; or it is both if you like. An affordance cuts across the dichotomy of subjective–objective” (p. 129). Taken together, developing sensorimotor relationship allow for sensorimotor mastery (Noë, 2004), or what in musical, everyday language may be referred to as virtuosity.

## Educational implications and concluding remarks

Viewing musical practice through the lens of affordances implicate an emphasis on creativity and exploration (Folkestad, 1996). With this follows a focus on agency since not all affordances must be realised (Stoffregen, 2003). Also imagination plays a crucial role (Van Dijk and Rietveld, 2020). Here, the learning environment is of importance. An open and safe environment will probably promote an explorative approach to learning, one which allows for affordances to emerge through open-ended interaction. The reverse scenario is equally likely – that a more determinate environment invites less imagination (van Dijk and Rietveld, 2020). In line with this, the focus on musical instruments of the present article must be seen as one piece of a wider creative ecology (Clarke, 2020). What might seem like peripheral activities of music learning might over time have profound consequences in terms of affordances. Beyond practice, there are a number of ways through which a musician may develop the sensorimotor relationship with their instrument. As noted above, this can mean alterations of the instrument end of the continuum, such as changes of the spatial layout (e.g., retuning, remodelling of keywork) and the sound-producing system (e.g., different strings, changing the bore of the instrument, preparations). These alterations have the potential to open up for explorations of new affordances of the instrument (DeSousa, 2017; Tullberg, 2021). Also, practising another instrument may impact the perception of the own instrument, as in the above example of piano playing. The same potential exists in learning music theory, through which conceptual knowledge may be transferred to spatial networks. Experience of learning other music traditions can reveal habitual ways of interacting with the instrument. Such awareness can direct attention to aspects of musicianship that previously were not available for reflection, and thus be the first step towards change, if so desired.

One way to operationalise these kinds of learning processes is through so called constraints-led approach, which has been applied in sports to some extent, for example in the learning lab at Southampton football club (see also Brymer and Renshaw, 2010). An example of such approach within musical practice is described by Slater (2020). By altering the constraints of the performance context, he is creating conditions through which new affordances can be explored. I think that the ecological approach within music research has gained momentum and that a joint forum for discussions and exchange of ideas on how the theory can be applied in practice would be beneficial.

I hope the path suggested in this paper can contribute to such a process. In essence, I have argued for a relational interpretation of affordances based on sensorimotor interaction with the instrument. Focusing on small-scale affordances, four conceptual components have been discussed above. (i) Even from a zoomed-in perspective, the musical niche is a crucial aspect, since it defines genre-specific conditions framing the musical practice. Among central aspects of the musical niche is the acoustic dimension as it is omnipresent and directly influencing the affordances of any musical event. (ii) There is a spatiality in all instruments and playing will cause spatial networks to emerge. (iii) These spatial networks are one essential part of the sensorimotor relationship, taken as the transactional space (Alessandroni and Malafouris, 2022). In line with MET, this sensorimotor relationship cannot be reduced to isolated parts, but is one cognitive system. (iv) From this follows an understanding of affordances as amodal. The sensorimotor relationship involves the whole body and its attunement to the situation (including abstract thought). Thus, it is not fruitful to seek to situate affordances in singular perceptual senses.

Moving forward, progressive theoretical work is necessary. Also, empirical accounts are crucial in order to make visible other conceptual components that needs to be considered in detail, and to more fully explore the ones highlighted in this article. Ethnographic projects have a potential to investigate the particular conditions in which musicians are working and thereby reveal structures of musical niches. Through inspiration from ethnomusicological work, such projects need to embrace the value pluralism (Alperson, 2008) that underly and inform musical practice.

The sensorimotor relationship, including spatial networks, can be further explored through variety of methods. Some of these, such as motion capture, naturally gravitate towards a laboratory setting. Nevertheless, taking the musical niche into consideration in the analysis, the results can be interpreted through an ecological lens. Furthermore, researcher-musicians and educators can contribute by autoethnographies and phenomenological explorations of their craft. Such first-person accounts have the potential to inform our understanding of perceptual and cognitive processes, hard to access from a third-person perspective. Collaborative research on and through musical practice, such as co-operative inquiry can contribute by exploring the diversity of experiences that exist even within a group of musicians playing the same instrument and working within the same genre.

These examples form a path in which empirical findings can feed back into a robust theoretical framework and conceptual clarity can guide educational progression.

## Data availability statement

The raw data supporting the conclusions of this article will be made available by the authors, without undue reservation.

## Ethics statement

Ethical review and approval was not required for the study on human participants in accordance with the local legislation and institutional requirements. Written informed consent for participation was not required for this study in accordance with the national legislation and the institutional requirements. Written informed consent was obtained from the individual(s) for the publication of any potentially identifiable images or data included in this article.

## Author contributions

The author confirms being the sole contributor of this work and has approved it for publication.

## Funding

This research was funded by the Faculty of Fine and Performing Arts, Lund University.

## Conflict of interest

The author declares that the research was conducted in the absence of any commercial or financial relationships that could be construed as a potential conflict of interest.

## Publisher’s note

All claims expressed in this article are solely those of the authors and do not necessarily represent those of their affiliated organizations, or those of the publisher, the editors and the reviewers. Any product that may be evaluated in this article, or claim that may be made by its manufacturer, is not guaranteed or endorsed by the publisher.

## Footnotes

^1^The interwoven relationship is referred to in slightly different ways: "perception-action interrelationship" (Michaels and Carello, 1981, p. 48); "perceiving-acting cycle" (Shaw, 2001, p. 296); "action/perception loop" (Östersjö, 2008, p. 78); and "perception-action coupling" (Warren Jr., 1990, p. 33).

^2^See for example Warren Jr.’s (1984) classic experiment with stair-case raiser height.

^3^Before him, similar ideas were presented by phenomenological thinkers such as Husserl (Jorba, 2020). Especially Merleau-Ponty’s (1945/2013) work on perception and the lived body has been combined with Gibson’s ideas. Examples of such approach within music research include Kim’s (2020) analysis of the process of learning to play a musical instrument and Nilsson’s (2011) artistic dissertation on improvisation on digital instruments.

^4^While enactivist theorists share the general concerns with Gibson (1979/2015) – the rejection of cognitivist perspectives – some of them are further apart from his work than others and (see Heras-Escribano, 2019; Heft, 2020; Alessandroni and Malafouris, 2022). Convergencies and divergencies between the ecological approach and enactivism has recently been explored in depth in a Frontier’s special topic (see the editorial article by McGann et al., 2020). As the editors note, taking the concept of affordances as locus point can further the theoretical debate: "In several papers here, affordances are deployed as a lens to bring certain points of contact between the ecological and enactive approaches into focus and examine them" (McGann et al., 2020, p. 3). Although this wider theoretical debate is beyond the scope of the present article, this paper is one example of how the ecological approach and enactivist theory can complement each other.

^5^In line with Gibson’s (1979/2015) notion of *ecological niche*, a musical niche does include, but is not limited to, physical space. For example, a music academy may be hosting several genres. Although residing inside the same building and being embraced by the same organization, various genres come with significant differences regarding the above conditions.

^6^Spivacke (1936) anticipated the research interest for coupling between acoustic space and musical stylistic development. Later musicological research along this line includes a study on how early Western art music and church architecture developed together (Navarro et al., 2009). See also Aletta and Kang (2020) for examples of research on historical acoustics.

^7^A rather drastic example of how stylistic development is constrained by the surrounding acoustics is *kulning*, herding calls found in Swedish traditional vocal music (Rosenberg et al., 2022). Acoustic characteristics that arose due to the desire to project sound over large distances remains as integral aspects of kulning, even when the practice is removed from its previous function and context, and instead performed on stage or in recording studios. In short, genre aesthetics develop in synergy with the acoustical spaces of the musical niche in a continuous process.

^8^Taking the surrounding space as an integral part of the instrument may seem counter intuitive as traditional organology treats instruments as sounding objects and rarely considers the acoustic environment. This line of research is probably best illustrated by Hornbostel and von Sachs’ (1914/1961) well-known classification system. Here, instruments are classified according to their sound producing mechanism. With its focus on the instrument itself, and isolated from the musical context, acoustic research carried out on musical instruments can be seen as an extension of this perspective. See Bijsterveld and Peters (2010) for a discussion on interdisciplinary potentials existing between the fields of organology and science and technology studies.

^9^Bielawski’s (1979) model can also lead to studies on control functions of musical instruments, which are in line with a dispositional view of affordances. Such mapping systems are generally not descriptions from "a performer’s point of view. Rather, the aim is to contribute to an analytical framework that may be used to describe general properties of instrument control" (Kvifte, 2008b, p. 355).

^10^Musical gestures are more fully explored by Godøy and Leman (2010).

^11^There are important differences between idiomaticity and spatial networks. While idiomaticity generally is understood in terms of instrument-specific movements or as musical conventions typical to a genre, spatial networks are not inherent properties of the instrument, nor a disposition of the musician. Instead, spatial networks emerge through the continuous interaction between the two.

^12^See also Hamilton and Duke (2020) regarding the relationship a co-development between performance level, motor behavior and perceptual skill.

^13^Clarke (2020) provides such an example through a description of guitarist Derek Bailey’s hand problems, drastically altering his ability to utilize the spatial networks.

^14^See Jorba (2020) for a discussion on Husserl’s notion of horizons and genuine and non-genuine presentations.

^15^See Magri (2019) for a discussion on the role of habits and attention with regards to a conceptualisation on affordances.
